# Thumbnail Tensor—A Method for Multidimensional Data Streams Clustering with an Efficient Tensor Subspace Model in the Scale-Space †

**DOI:** 10.3390/s19194088

**Published:** 2019-09-21

**Authors:** Bogusław Cyganek

**Affiliations:** Department of Electronics, Faculty of Computer Science, Electronics and Telecommunications, AGH University of Science and Technology, Krakow 30-059, Poland; cyganek@agh.edu.pl; Tel.: +48-12-617-3625

**Keywords:** tensor change detection, scale-space tensor decomposition, thumbnail tensor, video shot detection, orthogonal tensor subspaces, higher-order singular value decomposition

## Abstract

In this paper an efficient method for signal change detection in multidimensional data streams is proposed. A novel tensor model is suggested for input signal representation and analysis. The model is built from a part of the multidimensional stream by construction of the representing orthogonal tensor subspaces, computed with the higher-order singular value decomposition (HOSVD). Parts of the input data stream from successive time windows are then compared with the model, which is either updated or rebuilt, depending on the result of the proposed statistical inference rule. Due to processing of the input signal tensor in the scale-space, the thumbnail like output is obtained. Because of this, the method is called a thumbnail tensor. The method was experimentally verified on annotated video databases and on real underwater sequences. The results show a significant improvement over other methods both in terms of accuracy as well as in speed of operation time.

## 1. Introduction

Change detection in signal streams aims at finding time stamps, which correspond to signal variation as defined by some measures. In the result, the input stream is clustered into chunks that have minimized inter- and maximized intra-scatter, respectively. Moreover, the selected parts of the signal chunks can be used as a summary of the stream or, due to their inter-coherency, they can be efficiently compressed. Applications of such clusterings are ample in various domains related to signal processing. However, especially for the multi-dimensional signals, the task is not an easy one. Signal dimensionality, noise, as well as volume and speed of incoming data mean that the most successive methods are those that are highly adapted to the type of the processed signal. For example, in the case of video stream analysis, the majority of the efficient methods operate with specific image features, such as color, texture, sparse descriptors, etc. [1,2,3]. However, with a growing number of various sensors and measurements there is a need for more general methods that can operate with any type of signals. Such an approach, which relies on signal representation and analysis with tensor algebra, is presented in this paper. The method does not rely on any specific signal features and can operate with a variety of sensor measurements. However, there are not too many annotated multidimensional datasets that allow for reliable quantitative measurements. An exception are the available test video datasets [4]. Therefore, in this work we focus on the evaluation of shot detection in video streams.

The proposed method operates as follows. First, a part of the input multidimensional stream is extracted from the input stream. It is then converted to a tensor representation from which an orthogonal tensor subspace (OTS) is computed which constitutes our tensor model. The tensor model is computed with higher-order singular value decomposition (HOSVD). Then, during operation, tensors constructed from the successive chunks of the input signal are compared with this model. In effect, the model is either updated or it is rebuilt, depending on the statistical inference rule. However, such computations with tensors in their original size can be prohibitive even for the moderately low-dimensional signals. Therefore, in this paper a novel tensor preprocessing is put forward, which aims at minimizing tensor size, leaving as much of information as possible. Because of this, the method operates with much smaller tensors and, therefore, it is called a thumbnail tensor method. For this purpose, the two tensor transformations were verified. The first is tensor randomization, which consists of random drawing of selected tensor indices in order to lower its dimensions [5]. The second is based on the tensor scale-space [6]. In this approach, the frames are low-pass filtered and then processing takes place in the coarser scale. Despite a lower amount of data to process, this also makes the algorithm focus on upper-level information rather than on details. Because of this, and due to the application of the fast algorithm for tensor model updating, an order of magnitude speed up was obtained as compared with our previous work [7]. More importantly, with these techniques the method accuracy has also been greatly improved.

The presented method is an extended version of the method proposed in [7]. The record of this research is as follows. Following the tensor clustering method proposed by Sun et al. [8,9], in our work [10] a method based on the best rank-R (Tucker) tensor decomposition and the statistical inference was put forth. However, although reliable results were obtained, the maximal speed of this method did not exceed 3-4 frames per second. After further research, an idea of the video clustering into coherent clusters, which are bordered by signal shots, followed by the cluster compression with the best rank-R tensor decomposition, was outlined in our work [10]. Preliminary experiments also showed good results of this approach. Based on experience gained when working on the above projects, a question arose on a possible performance improvement, which could be obtained with other tensor decompositions. As a result, in the work of [7] a method based on HOSVD tensor decomposition was proposed. HOSVD, being simpler than the best rank-R decomposition, was used in our original publication [10]. However, HOSVD endowed with the fast model build algorithm, caught up with the accuracy of the method in [10], improving its performance at the same time. In the extended version, presented in this paper, significant improvements in accuracy and performance were gained, mostly due to the tensor size reduction method and parallel implementation. 

Also, in this work we significantly enlarged the experimentation framework with underwater images. These sequences were recorded in the artificial lake Zakrzowek (Poland), on behalf of the project for autonomous underwater object search. The sequences were then hand annotated to provide the representative keyframes. Computed with the proposed method, very interesting and highly accurate results were obtained, as will be presented in the experimental part in Section 5.

The work is organized as follows. Section 2 contains an overview of the research on shot detection in video streams, which we refer to in the experimental part. In Section 3 a general architecture of the proposed method is presented. On the other hand, Section 4 and its sub-sections contain basic descriptions of tensor algebra, the HOSVD tensor decomposition, as well as the proposed efficient algorithm for tensor model construction, respectively. In Section 5 the experimental results are presented. The paper ends with conclusions in Section 6.

## 2. An Overview of Shot Detection in Video Streams 

As already mentioned, the main experimental workbench for the presented method constitute color video signals. More specifically, for comparison with other methods the annotated VSUMM database was used, as will be presented in Section 5. It contains videos from the Open Video Project, in which video shots were annotated by human volunteers [11,12]. Therefore, in this section a short introduction and overview to the problem of abrupt change, or shot detection, is presented. Detection of abrupt signal changes in a video stream leads to its clustering into chunks. In the video processing community signal shots are usually assigned into one of the following categories [10,13]:

1. Hard cuts—an abrupt change of a content;
2. Soft cuts—a gradual change of a content;

The latter can further be separated into the following two groups:

1. Fade in/out—a new scene gradually appears or disappears from the current image;
2. Dissolving—a current shot fades out whereas the incoming one fades in.

A great majority of the video shot detection methods rely on the extraction of specific feature and subsequent data clustering and classification [2,3,14,15]. In this respect, the paper by Asghar et al. [16] contains a survey of video indexing. Also, the methods of temporal video segmentation and keyframe detection are discussed therein. On the other hand, a method for construction of a video abstraction is described in the work by Truong and Venkatesh [17]. An overview of the multi-view video summarization algorithms is presented in the paper by Fu et al. [18]. Efficient video summarization and retrieval tools are also discussed by Valdes and Martinez [19]. 

An interesting survey on video scene detection can be found in the work by Fabro and Böszörmenyi [20]. They classify the video segmentation methods into seven groups, depending on the low-level features used for the segmentation. The cited groups are as follows: the visual-based, audio-based, text-based, audio-visual, visual-textual, audio-textual and hybrid segmentations.

A unified scheme of shot boundary detection and anchor shot detection in news video story parsing is presented in the paper by Lee et al. [21]. Their method is based on a singular value decomposition, and the Kernel-ART method. On the other hand, DeMenthon et al. presented a video summarization method based on the curve simplification [22]. In this approach, a video sequence is represented as a trajectory curve embedded in a high dimensional feature space. It is then analyzed with the binary curve-splitting algorithm. This way partitioned videos are represented with the tree data structures. On the other hand, Mundur et al. [23] proposed another approach to video summarization. In their method, keyframe based video summarization is computed with Delaunay clustering [24]. A STIMO method storyboard creation from moving videos for the web scenario is proposed in the system by Furini et al. [25]. Their proposed algorithm is based on a fast clustering that selects the most representative video content using color distribution in the Hue-Saturation-Value (HSV) color space, computed on a frame-by-frame basis.

De Avila et al. suggested the already mentioned VSUMM method [11]. Their approach is based on the computation of color histograms from the video frames. These are then clustered with the k-means method. For each cluster, a frame closest to the cluster center is then chosen. This is the keyframe that represents a given slice of a video. De Avila et al. also proposed a method of video static summaries evaluation which is used for method comparison. Also in this paper we follow their proposed evaluation strategy with the help of the user annotations available from the Internet [12]. 

Color histograms for video summarization have been suggested in the method by Cayllahua-Cahuina et al. [26]. In this approach, 3D histograms of 16 × 16 × 16 bins are calculated directly from the RGB image representation. In the result, 4096 dimensional vectors are obtained. These are further compressed with the PCA method. In the next step, two clustering algorithms are launched. Fuzzy-ART is used for the determination of a number of clusters. After this, Fuzzy C-Means performs frame clustering from the color histogram features. However, using only color information is not enough to obtain the satisfactory results, as shown in [26].

In the papers by Medentzidou and Kotropoulos, video summarization methods, based on shot boundary detection with penalized contrast, are put forth [27]. These approaches also rely on color analysis, however, this time in the HSV color space. The mean of the hue component is used as a main indicator of a change in the video. Then, video segments are extracted and represented with a linear model. As reported, the method obtains results comparable to the VSUMM method by de Avila et al. [11].

The next video summarization method, called VSCAN, was proposed by Mahmoud et al. [28]. In this approach, a modified density-based spatial DBSCAN-like clustering method is used. However, once again, video summarization is based entirely on color and texture feature processing.

The concepts of the data stream analysis, concept drift detection, as well as data classification in data streams are also discussed in this paper. In this respect, the book by Gama [1] or the paper by Krawczyk et al. [3], can be recommended as further introductions to this subject.

On the other hand, a short introduction to the tensor algebra and tensor decomposition methods, used in the proposed methods, is contained in Section 4.

## 3. A Framework for Multidimensional Data Stream Clustering

**Figure 1:**
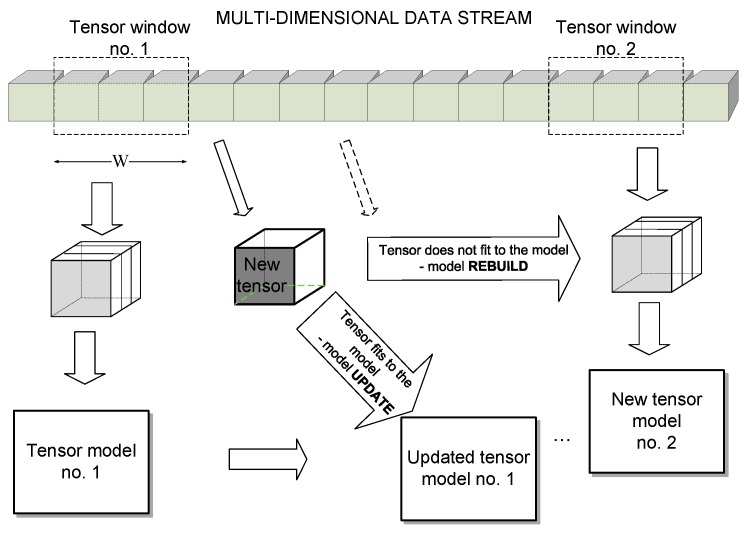
Architecture of the multi-dimensional data stream analysis in the proposed tensor framework. A window of size *W* is selected to build the tensor model. Further chunks of data are compared with this model. If data fits the model, then the model is updated, otherwise the model is rebuilt starting at the current position.

An architecture of the multi-dimensional data stream processing in the proposed tensor framework is depicted in Figure 1. We assume that the input stream may consist of potentially infinite series of *L*-dimensional signals, which can be represented as tensors. From these, at a given time stamp, a window of a fixed size *W* is selected from which a model tensor is computed, as will be discussed. Then, each incoming data tensor is checked to fit to this model. If it does, then the model is updated, as will be discussed. Otherwise, the model is rebuilt starting at the current data position, and the whole process is repeated.

As alluded to previously, a similar scheme based on the best rank-R tensor decomposition has been proposed in our work [13]. However, this model is computationally demanding; The algorithm is iterative and requires tensor decomposition in all its dimensions. Therefore, although our previous method produced good results, its operation time allows for processing of only up to three color frames per second. In this paper we suggest a simpler tensor model, which is based on OTS computation with HOSVD of the reduced input tensors. By contrast with the best rank-R algorithm, OTS requires only one solution to the eigenvalue problem. Because of this and as a result of the proposed representation, it is computed from a symmetric matrix of a small size *W* × *W*, as will be discussed. Furthermore, the matrix is smaller due to reduction of the input data. All these, as well as application of the fast eigenvalue computation with an automatic selection of the leading eigenvectors, result in much better accuracy. Also, an order of magnitude speed up was achieved as compared to other tensor-based methods. In the next sections, details of the computational steps in Figure 1 are presented.

## 4. Construction of the Orthogonal Tensor Subspace (OTS)-Based Model

Tensors in processing of multi-dimensional data offer many advantages compared to the vector-based methods. Most importantly, in the tensor domain the neighbor relations among elements are retained, whereas a separate dimension of a tensor represents each degree of freedom. Another advantage is that tensor methods can work with any type of signal since no specific features are assumed. In the next sections, we present a short introduction to signal representation and processing with tensor based methods. Also details of our suggested tensor model and its updating scheme are presented. Further information on tensor processing can be referred to in literature [6,29,30,31,32].

### 4.1. Higher-Order Singular Value Decomposition (HOSVD) for Data Stream Analysis

Since the experimental results presented in this paper are related to tensors composed from the 3D color video frames, with no loss of generality, a further analysis in this section is constrained to the 4D tensors.

Tensor algebra found its place in 20th-century physics as a convenient tool for formulation of the general relativity. In this respect, the distinctive properties of tensors are their transformation rules, which precisely describe change of tensor components in respect to a change of the coordinate system [6]. However, since then other definitions of tensors have been formulated—as the multi-linear maps and as the multidimensional arrays of real numbers, respectively. With these new interpretations, tensors found broader applications in psychometrics, chemometrics, data science, geophysics, mechanics analysis, as well as in computer vision, pattern recognition and graphics, just to name a few [6,29,31,32,33]. We also follow this newer interpretation of tensors. Therefore, it is assumed that a 4D tensor can be represented as a four-dimensional array of real values, that is (tensors are written with calligraphic letters, while for matrices and vectors the bold font is used).

(1) $$T\in {ℜ}^{N_{1}\times N_{2}\times N_{3}\times N_{4}}$$

where *N_j_* stands for a *j*-th dimension, for 1 ≤ *j* ≤ 4. On the other hand, with no loss of information, each tensor can be unanimously represented in a matrix representation. Such representation, known as a tensor flattening, will be extensively used in the algorithms presented in further part of this paper. In this approach, a flattening in the *j*-th dimension is defined as the following matrix:

(2) $$T_{\left(j\right)}\in {ℜ}^{N_{j}\times \left(\ldots N_{j-1}N_{j+1}\ldots \right)}$$

In other words, tensor flattening is obtained from a tensor T by selecting its *j*-th dimension, to become a row dimension of the matrix **T**_(*j*)_. On the other hand, a product of all other indices constitutes a column dimension of the matrix **T**_(*j*)_. 

In further derivations the *k*-th modal product of a tensor $T\in {ℜ}^{N_{1}\times \ldots \times N_{4}}$ and a matrix $M\in {ℜ}^{Q_{}\times N_{k}}$ is employed. The result of this product is a tensor $S\in {ℜ}^{N_{1}\times \ldots N_{k-1}\times Q\times N_{k+1}\times \ldots N_{4}}$, defined as follows:

(3) $$S_{n_{1}\ldots n_{k-1}qn_{k+1}\ldots n_{4}}={\left(T\times_{k}M\right)}_{n_{1}\ldots n_{k-1}qn_{k+1}\ldots n_{4}}=\sum_{n_{k}=1}^{N_{k}}t_{n_{1}\ldots n_{k-1}n_{k}n_{k+1}\ldots n_{4}}m_{qn_{k}}.$$

However, what is really interesting from the analytical point of view, are tensor decompositions. In this paper the HOSVD decomposition will be used. Namely, considering the tensor properties (1)–(3), the HOSVD decomposition of a tensor $T\in {ℜ}^{N_{1}\times N_{2}\times N_{3}\times N_{4}}$ is defined as follows [6,34,35,36,37]:

(4) $$T=Z\times_{1}S_{1}\times_{2}S_{2}\times_{3}S_{3}\times_{4}S_{4}$$

where **S**_k_ stands for a unitary mode matrix of dimensions *N_k_* × *N_k_*, and $Z\in {ℜ}^{N_{1}\times N_{2}\times N_{3}\times N_{4}}$ is a core tensor {XE “tensor:core”} of the same dimensions as T. Furthermore, it can be shown that the core tensor Z fulfills the following properties:

1. Two sub-tensors $Z_{n_{k}=a}$ and $Z_{n_{k}=b}$, obtained by fixing the *n_k_* index to *a*, or *b*, are orthogonal, that is, for all possible values of *k* for which *a* ≠ *b* the following holds:
  (5) $$Z_{n_{k}=a}\cdot Z_{n_{k}=b}=0$$
2. All sub-tensors can be ordered according to their Frobenius norms:
  (6) $$\left\|Z_{n_{k}=1}\right\|\geq \left\|Z_{n_{k}=2}\right\|\geq \ldots \geq \left\|Z_{n_{k}=N_{P}}\right\|\geq 0$$

In the framework put forth in [13], and also in this paper, the input tensor T is composed of a series of 3D frame-tensors ${ℱ}_{w}$, for 1 ≤ *w* ≤ *W*. That is, the input tensor is constructed as follows:

(7) $$T=\left[{ℱ}_{1}|{ℱ}_{2}|\ldots |{ℱ}_{W}\right]$$

However, in this work size of the input tensor is reduced by the two methods:

1. Randomization, by means of a random selection of rows and columns. This is based on the Mersenne uniform twister in order to achieve tensor of given lower dimensions. As shown in recent works by Halko et al. [5], as well as by Zhou et al. [38], such randomization simplifies tensor processing and, even more importantly, allows for the discovery of the low-rank structure in huge tensors.
2. The scale-space approach in which the input signal is low-pass filtered and down-sampled to the given lower dimensions. Such a strategy has been applied in the famous SIFT detector [39] or for object detection in e.g. our previous works [14,40].

**Figure 2:**
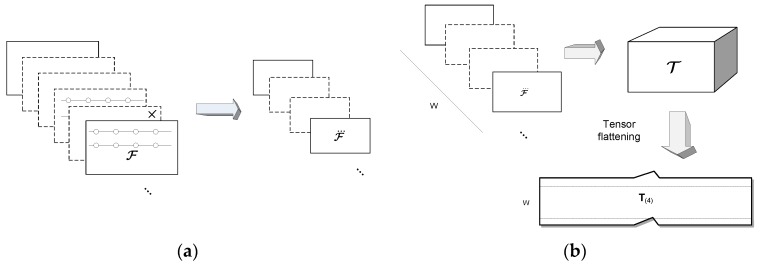
The input stream consists of potentially infinite series of D-dimensional tensors. This is reduced in all (or selected) dimensions either by the uniform randomization or by the low-pass filtering to the coarser scale-space level (**a**). From these, a window of a fixed size *W* is selected from which a model tensor is constructed. The orthogonal tensor subspace (OTS) model is computed from one tensor flattening alongside its last dimension. It is easy to observe that each data from the series constitutes one row in this flattening. The order of flattening is irrelevant if kept consistent among all tensors (**b**).

In effect, a reduced version of the input tensor is obtained, as follows:

(8) $$T=\left[{\dddot{ℱ}}_{1}|\ldots |{\dddot{ℱ}}_{i+D}|\ldots |{\dddot{ℱ}}_{W}\right]$$

where ${\dddot{ℱ}}_{i}$ denotes either reduced version of the original tensor ${ℱ}_{i}$. This process is depicted in Figure 2a. It should be noted however, that in experiments on video signals the second method, that is scale-space decimation, provided better results by 1–4%, compared to the tensor randomization. This can be caused by natural characteristics of the visual signals in which neighboring pixels are highly correlated. Therefore, in further considerations we refer to the second method of tensor reduction. However, the randomized version can be a beneficiary in the cases with not so strong correlation of neighboring elements [5,38]. Moreover, in our experiments the same decimation coefficient *D*, in order 0.2–1.0, was used to uniformly reduce tensor size in all dimensions. 

Subsequent construction of the tensor T is depicted in Figure 2b. T can be now decomposed with the HOSVD decomposition to build an OTS. OTS serves as a model to the reduced tensor window *W*. In this respect we follow the idea of Savas and Eldén, originally presented in [41], then followed in [7]. It is easy to notice, that the simple re-arrangement of (4) yields:

(9) $$T=\underset{{ℬ}_{w}}{\underset{︸}{\left(Z\times_{1}S_{1}\times_{2}S_{2}\times_{3}S_{3}\right)}}\times_{4}S_{4}$$

where owning to the condition (5), tensors ${ℬ}_{w}$ for 1 ≤ w ≤ W, are orthogonal. In other words, their product gives 0. Equation (9) can be further written as follows:

(10) $$T=\sum_{w=1}^{W}\left({ℬ}_{w}\times_{4}s_{4}^{w}\right)$$

where vectors $s_{4}^{w}$ denote columns of the unitary matrix **S**_4_. Because each tensor ${ℬ}_{w}$ is three-dimensional, then ×_4_ denotes the outer product of each 3D tensor and a vector $s_{4}^{w}$, as defined in (3).

**Figure 3:**
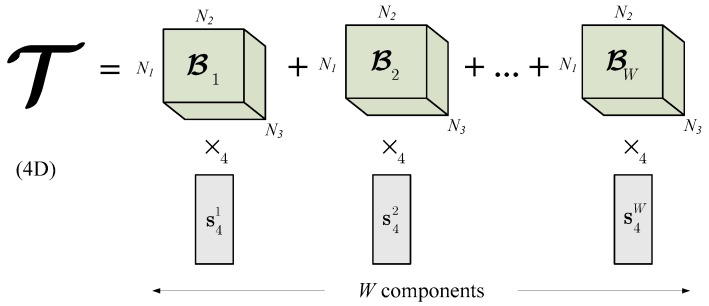
An example of the OTS of a 4D model tensor spanned by a series of orthogonal 3D base tensors. The OTS represents a model of a window of W input data tensors. A distance of a tensor to the model is computed as its projection onto the OTS.

This way, from the tensor T the OTS is constructed, as visualized in Figure 3. In other words, the OTS constitutes a model. The reason of constructing the OTS is its ability to represent the series of *W* frames, as well as to introduce a distance of an incoming data (tensor) to that space. This property will be used to check a fitness measure of each frame to the model, as will be discussed.

For a given reduced tensor $\dddot{ℱ}$, its distance to the model represented by a series of base tensors $\left\{{ℬ}_{w}\right\}$, is computed as a sum of squared inner products, as follows [7]

(11) $$R=\sum_{w=1}^{W}{\langle {ℬ}_{w}^{},\dddot{ℱ}\rangle }^{2}$$

Tensors used in the above formula need to be normalized. Value of (11) will be used to assess model consistency. That is, values of *R* are computed for all frames belonging to the model. Then *R* is computed for each new frame to tell its consistence with the model, as will be discussed.

### 4.2. Efficient Computation of the Orthogonal Tensor Subspaces

Since efficient computation of the base tensors ${ℬ}_{w}$ from the input signal is essential for operation of the method, in this section we propose an effective algorithm. Practically, ${ℬ}_{w}$ can be simply computed after rearrangement of Equation (7), as follows [7,42]:

(12) $$T\times_{4}S_{4}^{T}=Z\times_{1}S_{1}\times_{2}S_{2}\times_{3}S_{3}={ℬ}_{w}$$

Thus, to compute ${ℬ}_{w}$ it is sufficient to compute only the mode matrix **S**_4_. It can be computed from the SVD decomposition of the flattened matrix **T**_(4)_, that is:

(13) $$T_{\left(4\right)}=S_{4}V_{4}D_{4}^{T}$$

However, **T**_(4)_ is large, with a number of rows equal to *W* and the number of columns being the product of its dimensions 1–3. For example, for a color video this is a total number of pixels in the input frames times three color channels. To overcome this problem, both sides of (13) can be multiplied by $T_{\left(4\right)}^{T}$, to obtain the following:

(14) $$T_{\left(4\right)}T_{\left(4\right)}^{T}=S_{4}V_{4}^{2}S_{4}^{T}$$

The above product $T_{\left(4\right)}T_{\left(4\right)}^{T}$ has dimensions of only *W* × *W*. Moreover, it is a symmetrical matrix. Owning to these properties, an effective fixed-point eigenvalue decomposition algorithm can used, as is described in the next section. What is also important is that the OTS model is computed only once and from a smaller matrix due to tensor processing in the scale-space (or randomization), as well as to the condition (14). Because of this, computation of the base tensors can be much faster compared with other tensor decomposition schemes. The above steps of the model building procedure are shown in Algorithm 1.


**Algorithm 1. Computation steps of the OTS tensor model building.**

**Input:**

**A finite partition of the multi-dimensional data stream from a window *W*;**

**Output:**
An orthogonal tensor subspace (OTS) represented with the base tensors ${ℬ}_{w}$;1.Fill the buffer with *W* input data and construct scale-space/randomized tensor T in (8);2.Construct the flattened matrix **T**_(4)_ of a tensor T;3.Compute the product $T_{\left(4\right)}T_{\left(4\right)}^{T}$;4.With Algorithm 3 compute the **S**_4_ as eigenvectors of the symmetric matrix $T_{\left(4\right)}T_{\left(4\right)}^{T}$ in (14);5.From (12) compute the bases ${ℬ}_{w}$;

### 4.3. Model Fitness Measure and Efficient Model Updating Scheme

As already pointed out, the measure *R* in (11) can be used to tell a distance of a tensor $\dddot{ℱ}$ to the OTS model represented by the basis $\left\{{ℬ}_{w}\right\}$. Values of *R* for the model frames, as well as for all other frames from the stream can be used for the statistical analysis of abrupt signal changes in the stream. However, instead of the absolute values of *R*, better results are obtained when the differences of ΔR are used for computations. That is, the following error function is defined as:

(15) $$\Delta R_{i}\equiv R_{i}-R_{i-1}$$

For proper detection of the shots with slowly changing content, the following drift measure is proposed [13]:

(16) $$\left\|\Delta R_{ℱ}-{\bar{R}}_{\Delta }\right\|<\;a\;\sigma_{\Delta }+b$$

where *a* is a multiplicative factor (3.0–4.0) and *b* is an additive component (0.2–2.5). The parameters ${\bar{R}}_{\Delta }$ and $\sigma_{\Delta }$ denote the mean and standard deviation computed from the differences of fit values in (15) for the model frames from (7), as follows:

(17) $${\bar{R}}_{\Delta }=\frac{1}{W}\sum_{w=1}^{W}R_{\Delta }{}_{w},\;\mathrm{and}\;\sigma_{\Delta }^{2}=\frac{1}{W-1}\sum_{w=1}^{W}{\left(R_{\Delta }{}_{w}-{\bar{R}}_{\Delta }\right)}^{2}.$$

Each new tensor $\dddot{ℱ}$ is checked to fit to the model in accordance with (17). If it does not fit, the model has to be rebuilt from a new set of frames, starting at the position of $\dddot{ℱ}$. However, to achieve robustness against some spurious signals, in the fitness algorithm it is required that a number *G* of consecutive frames divert from the model in order to start the model rebuild process.

**Figure 4:**
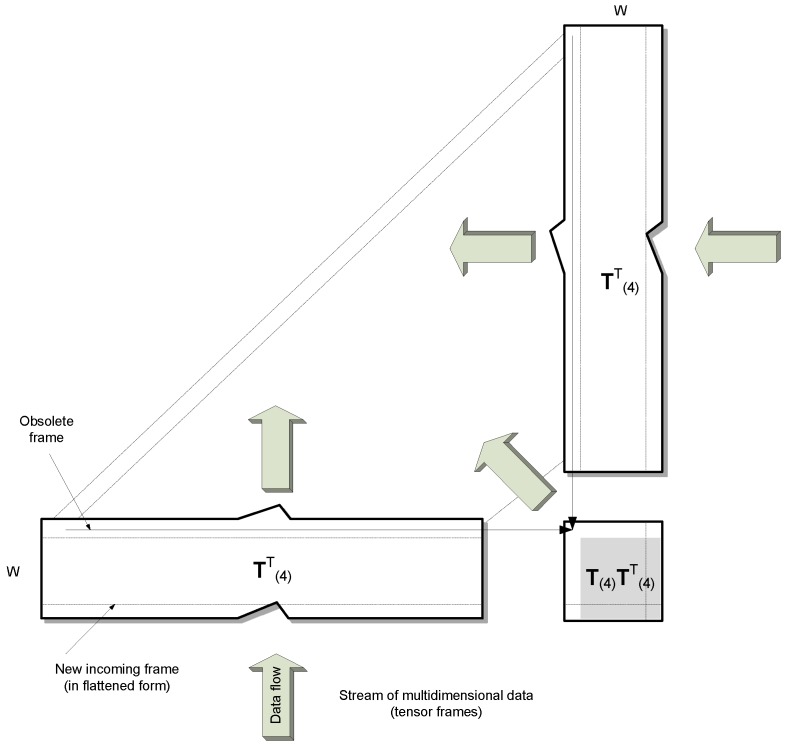
An update scheme for the flattened version **T**_(4)_ of the tensor model by a new frame. This is done by simple insertion of the new row and by obliterating of the oldest one in **T**_(4)_. In the product matrix $T_{\left(4\right)}T_{\left(4\right)}^{T}$ all values, except for one row and one column, can be reused as shown in Figure 5.

**Figure 5:**
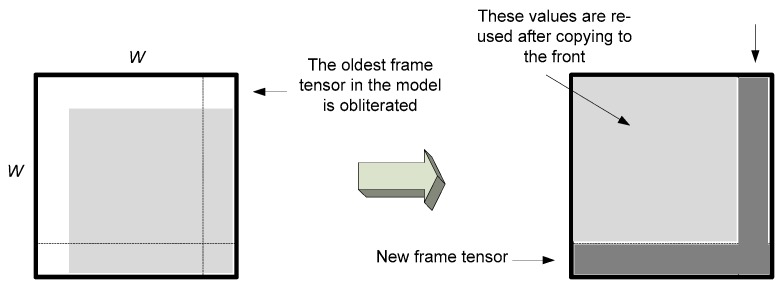
Efficient updating scheme of the product matrix $T_{\left(4\right)}T_{\left(4\right)}^{T}$.

On the other hand, if $\dddot{ℱ}$ fits to the model, the model needs only to be updated. Figure 4 depicts the proposed efficient method of updating of the flattened version **T**_(4)_ of the model tensor. This is done by simple insertion of *only* the new row and obliterating the oldest row in the **T**_(4)_ matrix. Consequently, in the product matrix $T_{\left(4\right)}T_{\left(4\right)}^{T}$ all values except one row and one column can be reused, as shown in Figure 5. The following steps describe the model update algorithm (Algorithm 2). 


**Algorithm 2. Tensor model updating algorithm.**

**Input:**
**New tensor frame**$\dddot{ℱ}$;
**Output:**
New model (bases ${ℬ}_{w}$);1.Shift data in **T**_(4)_ by one row up (Figure 4) and fill the last row with flattened version of $\dddot{ℱ}$;2.Shift all data in the old $T_{\left(4\right)}T_{\left(4\right)}^{T}$ matrix by one row up and to the left (Figure 5);3.Fill up the last row and right column in $T_{\left(4\right)}T_{\left(4\right)}^{T}$ with a product of the flattened $\dddot{ℱ}$ and all remaining (old) frames from **T**_(4)_;4.Perform steps 4 and 5 of the model build Algorithm 1;

In the above model updating algorithm the most time-consuming is step 3, which involves *W*-1 products of the tensor frames. On the other hand, the last step 4 is relatively fast and consumes the same amount of time as in the full model build step, since it requires a solution of the eigenvalue problem of a matrix of size *W* × *W*.

### 4.4. Efficient Computation of the Leading Eigenvectors

The computation steps in formula (14) involve only the positive real symmetric matrices. Therefore, it is possible to employ a faster algorithm than the general SVD decomposition. For this purpose, the so called fixed-point eigen-decomposition algorithm is proposed [42,43]. Algorithm 3 shows the key steps of this method. 


**Algorithm 3. An efficient computation of the *K* leading eigenvectors of a symmetric product matrix P.**

**Input:**
A real symmetric matrix **P;** A number *K* of expected eigenvectors: 1 ≤ *K* ≤ *rows*(**P**); A maximal number of iterations *i_max_*; An orthogonality threshold *ε*;
**Output:**
*K* leading eigenvectors of **P**;1.Randomly initialize $e_{0}^{\left(0\right)}$;2.Set *k* ← 0;3.Set *err* ← 2*ε*;4.**while***k* < *K*5.  Set *i* ← 1;6.  **while**
err>ε
**and**
*i* < *i_max_*7.    
$e_{k}^{\left(i\right)}\leftarrow P\;e_{k}^{\left(i-1\right)}$
8.      $e_{k}^{\left(i\right)}\leftarrow {e_{k}^{\left(i\right)}/\left\|e_{k}^{\left(i\right)}\right\|}_{2}$      ($e_{k}^{\left(i\right)}$ normalization)9.    $e_{k}^{\left(i\right)}\leftarrow e_{k}^{\left(i\right)}-\sum_{j=0}^{k-1}\left(e_{k}^{T\left(i\right)}e_{j}^{}\right)e_{j}^{}$ (Gram-Schmidt)10.$e_{k}^{\left(i\right)}\leftarrow {e_{k}^{\left(i\right)}/\left\|e_{k}^{\left(i\right)}\right\|}_{2}$          ($e_{k}^{\left(i\right)}$ normalization)11.
$err=\left|e_{k}^{T\left(i-1\right)}e_{k}^{\left(i\right)}-1\right|$
12.Set *i* ← *i* + 1

As shown in our previous work, application of this fast algorithm allows for even five times speed up. Further properties of this algorithm are discussed in the following publications [13,42,44]. 

### 4.5. Computation of the Leading Eigenvectors

The main idea behind finding the number of significant eigenvalues in decomposition (14) is based on the detection of a significant drop in the ordered series of eigenvalues λ_i_. This corresponds to an observation that the large eigenvalues correspond to variances of the latent variables, whereas small eigenvalues are usually due to noise. For this purpose a difference between logarithms of the largest one λ_1_ and λ_i_ and are used. In the first step, the reference slope *R_M_* of *M*−1 initial, that is, the largest eigenvalues λ_i_ is computed as follows:

(18) $$R_{M}=\frac{1}{M-1}\sum_{i=2}^{M}\left[\log\left(\lambda_{1}\right)-\log\left(\lambda_{i}\right)\right]$$

In the next step, each newly computed eigenvalue is compared to the *R_M_*, as follows:

(19) $$d=\{\begin{array}{ll} 1 & if\;\;\log\left(\lambda_{1}\right)-\log\left(\lambda_{i}\right)>\eta R_{M},\;\;for\;\;i>M\\ 0 & otherwise \end{array}$$

If *d* in (19) reaches 1, then the process of computing eigenvectors is immediately stopped. The above procedure of incremental computation is controlled by two parameters *M* and *η*. In our experiments, the best results were obtained for *M* fixed to 3, while *η* was set in the range 2–5.

## 5. Experimental Results

The method was implemented entirely in C++ in the Microsoft Visual 2017 IDE. For the basic tensor operations the DeRecLib library was employed [45]. The experiments were run on a computer with the Intel® i9-7960X CPU @ 2.80GHz, 64 GB RAM, and in the 64-bit Windows 10. 

As already mentioned, the proposed method can work with any type of signal of any finite dimensions, since no specific features are computed. However, it is not easy to find suitable test streams with ground truth annotations. Therefore, and to compare results with other works, for evaluation the VSUMM database was used. It contains 50 color videos from the Open Video Project [12,46]. The video sequences are of resolution 352 × 240 pixels, 30 fps, with duration in the range of 1 to 4 min, encoded in the MPEG-1 [4]. The total number of frames is 57,895. For each video in the VSUMM database there are annotated shots obtained by five human annotators.

For the qualitative evaluation, the parameters proposed by de Avila et al. [11,16], called Comparison of User Summaries were used. These are *CUS_A_* = *n_AU_*/*n_U_* and *CUS_E_* = ~*n_AU_*/*n_U_*, where *n_A_* denotes a number of matching keyframes from the automatic summary (AS) and the user annotated summary, ~*n_AU_* is the complement of this set (i.e., the frames that were not matched), while *n_U_* is a total number of keyframes from the user summary only (US). However, in other works the precision *P* and recall *R*, parameters are preferred, since they convey also information on the keyframes present in AS and not present in US or vice versa, as discussed by Mahmoud et al. [28,46]. As a tradeoff of the two, in many works the *F* measure is also used [28,47]. Such an approach has been also undertaken in our experiments. The above quantities are defined as follows:

(20) $$P=\frac{n_{AU}}{n_{A}},\;R=\frac{n_{AU}}{n_{U}},\;\mathrm{and}F=2\frac{PR}{P+R},$$

where *n_AU_* is a number of keyframes from the AS that match those from the US, *n_A_* is a number of total keyframes from the AS only, while *n_U_* from the US, respectively. 

**Figure 6:**
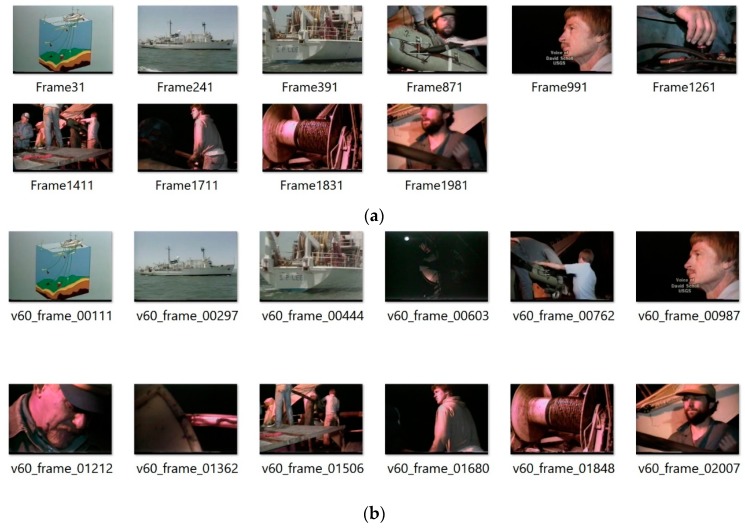
User selected images from the “The Voyage of the Lee, segment 05” sequence (**a**). These are compared with the thumbnails computed by our algorithm (**b**).

**Figure 7:**
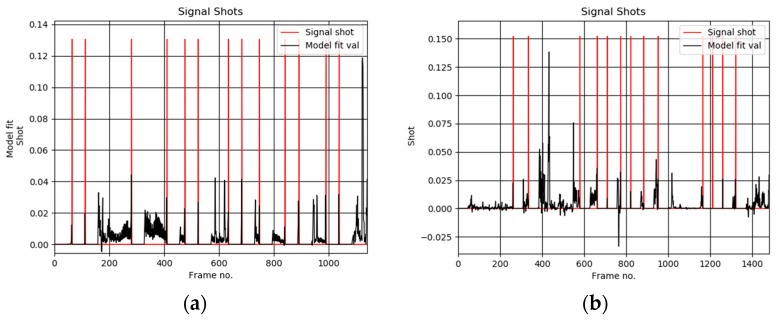

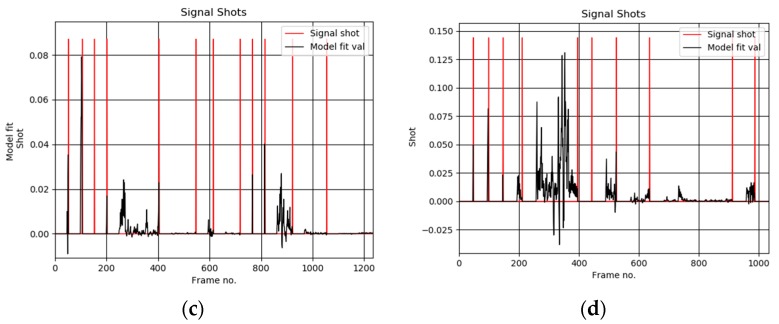
Plots of the detected scene shots computed by our method in the video sequence no 28 (**a**), 38 (**b**), 48 (**c**), and 58 (**d**) from the Open Video Database [4], respectively. Frames were decimated in space and time by a factor 0.33.

Figure 6a shows exemplary user selected keyframes from the test sequence “The Voyage of the Lee, segment 05”, from the Open Video Database [4]. These are compared with the thumbnails computed by our algorithm, as shown in Figure 6b. Based on such comparisons the average value of the accuracy parameter *F* was computed, as shown in Table 1. On the other hand, detailed values for each test sequence are provided in Table 2. Figure 7 show plots of the detected thumbnail tensors from the test sequences no 28 (also shown in Figure 6), 38, 48, and 58, respectively.

As shown in Table 1, the proposed method outperforms methods reported by other researchers, except for the VSCAN for which it performs equally well. Interestingly enough, our method outperforms other methods even if run on a monochromatic version of the input videos, as shown in the second row of Table 1. In this case its performance is only slightly worse compared to the case when operating with the fully dimensional signal. This is an interesting feature of the tensor-based methods—they easily scale to various dimensions of the input signal. On the other hand, the other methods, being tuned to specific features, such as color or texture, do not possess this feature and cannot run without the color information. At the same time, the proposed thumbnail tensor method is an order of magnitude faster than other tensor methods and the referenced methods for which timings were available. Detailed computational times of our method are presented in Table 3.

The important aspect of the group of tensor based methods is that, they do not put any specific assumptions on a type of the input signal. In other words, no specific statistical properties, nor specific features, are required. However, investigation of the method performance with other types of multi-dimensional signals is left for further research.

As alluded to previously, for size reduction of the input tensor, two methods were tested. The first of them is based on tensor randomization. However, the second one, which is based on construction of the scale-space and operation with the coarse scale, performed better for the color and monochrome video signals. Depending of a video sequence, the difference was in order of 1–4%. This is caused by high correlation of neighboring pixels in the video signals. However, the randomization methods have also high potential, especially when working with other types of signals. The randomized method gain on popularity due to recent achievements in matrix and tensor approximations and big data [5,38]. Investigation of tensor randomization in our framework is left for further research, especially when other types of signals will be available.

Table 4 contains values of the important parameters that control operation of the proposed thumbnail tensor method.

**Figure 8:**
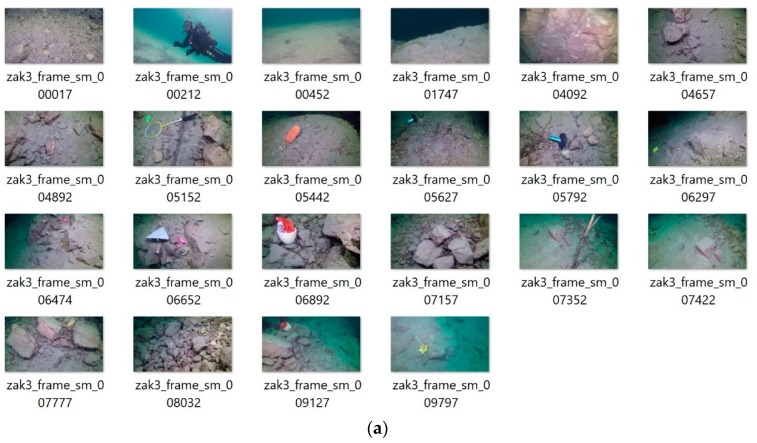

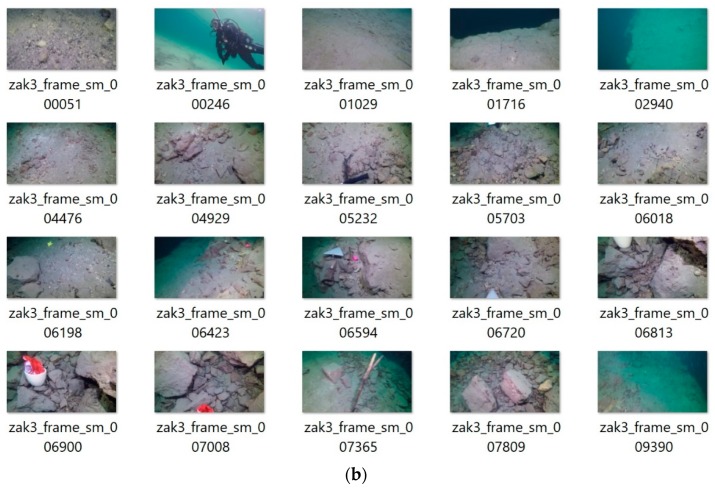
User-selected images from the “Zakrzowek No. 3” sequence (**a**). The representative frames are compared with the thumbnails computed by our algorithm (**b**). The best accuracy obtained 0.71, with the processing time 140 frames/s.

The proposed method was finally applied to another problem of object search in underwater video signals. In this experiment a number of human-made objects, such as a shoe, a trowel, a wheel, etc. where drawn in random places in the artificial lake (Zakrzówek, Poland). Then the footage was acquired which contains these, as well as thousands of natural objects, such as stones, plants, underwater hills, etc. There is a total of 10,845 frames in the video. From these, subjectively representative images with various objects were chosen. After that, the method was run with the same parameters as in the previous experiments. The user selected representative frames, as well as results of our method, are presented in Figure 8a,b, respectively. 

It can be noticed that in majority of cases our algorithm also detected the human made objects which protrude from the background. Its overall accuracy in this test was 0.71, with execution speed of 140 frames/s.

**Figure 9:**
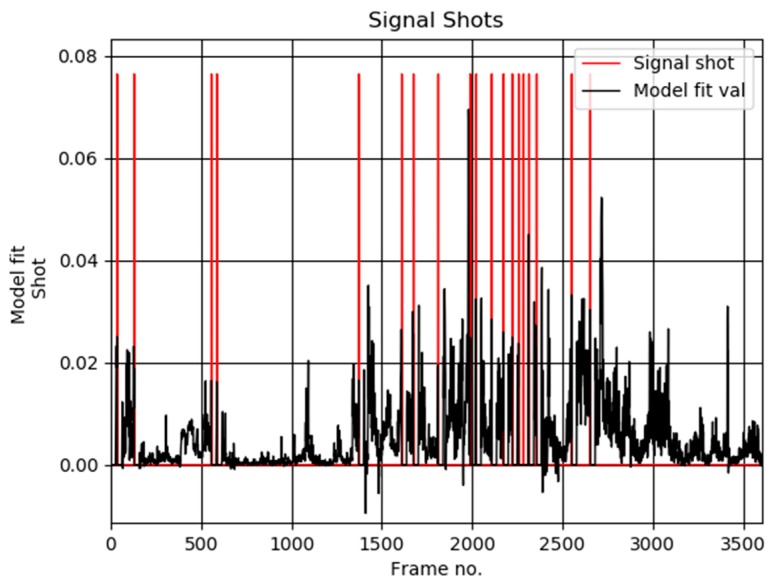
A plot of the detected scene shots in the underwater video sequence Zakrzowek No. 3. Frames decimated in space and time by a factor 0.33.

Figure 9 shows a plot of the detected scene shots computed by our method in the underwater video sequence Zakrzowek No. 3.

In all of the presented experiments accuracy has to be seen in the context of the highly subjective key frame choice by users. Our algorithm does not check if a similar scene was already observed, say a few shots beforehand, so for example the trowel is detected two times but in timely separated shots, that is frame 6663 vs. 9333 as shown in Figure 8b. Interestingly enough, almost all changes caused by the appearance of artificial objects were detected. On the other hand, there were not too many false alarms caused by changes of natural objects. This means that the method reacts well on abrupt changes caused by an ‘unusual’ object, which frequently is not very big but which is significantly different from the background. Such a feature is specific to human observers—it is much easier to detect something ‘unusual’ in the context, such as a shoe or a trowel in underwater scenery. 

## 6. Conclusions

In this paper an improved version of our previous work on the multi-dimensional signal streams clustering is presented. Stream clustering is done by searching for abrupt signal changes, based on the proposed tensor framework and the statistical inference rules. In the tensor framework, a multi-dimensional signal is modeled with the orthogonal tensor subspaces, computed with the HOSVD tensor decomposition. However, the main novelty presented in this paper is tensor size reduction by means of tensor randomization and scale-space filtering. Because of this, an order of magnitude speed up has been achieved as compared with the previous version of this method. Due to much smaller input tensors, the output comes in the form of the so called thumbnail tensors. Other contributions of this paper constitute the two efficient algorithms for tensor model construction and its update, respectively. These are based on the fast eigenvalue computation with the mechanism for an automatic choice of the important eigen-components. The method was tested with the Open Video Database that contains signal shots annotations. In terms of the achieved accuracy, the proposed method outperforms other methods presented in the literature, with only one performing equally well. The method was also tested on a real underwater sequence, in the task of scene change detection to facilitate detection of human made drawn objects. Despite the different physical properties of light propagation in water, our method performs equally well, achieving accuracy in the order of 71% with processing speed of 140 frames/s in software implementation.

Although tested on color video streams, the proposed algorithms are general in the sense that there are not any specific assumptions on dimensionality or type of the input signal. Therefore, the proposed framework can be applied to various types of multidimensional signals [48,49]. Also, the suggested construction of the orthogonal tensor subspaces can be used in many other classification and clustering frameworks which require the comparison of tensors. 

Further research will be focused on the connection of the signal-clustering method, proposed in this paper, with an efficient data-compression method based on tensor decompositions, as already outlined in one of our works [10]. The main idea is based on an observation that, due to the high inter coherence of the signal chunks, it is possible to obtain high compression ratio with no significant loss of precision in signal reconstruction. Also, the method needs to be tested with other types of multi-dimensional data streams. However, it is difficult to find annotated databases of multidimensional signals. Therefore, further research will be conducted towards the creation of databases containing annotated synthetic and real examples. 

## Figures and Tables

**Table 1:** Accuracy comparison with other methods.

Method	OV [22]	DT [23]	STIMO [25]	VSUMM [11]	VSCAN [28]	Best rank-R [13]	HOSVD [7]	HOSVD (This Paper)
*F*	0.67	0.61	0.65	0.72	**0.77**	0.73	0.73	**0.77**
*F-MONO*	NA	NA	NA	NA	NA	NA	0.71	0.76

**Table 2:** Accuracy of the tensor thumbnail method measured on the Open Video Project database. A total number of frames to process 57,895. Average accuracy 0.77. Total execution time 224 s.

Seq. No.	CUS_A_	CUS_E_	*P*	*R*	*F*
21	0.64	0.45	0.58	0.64	0.61
22	0.75	0.25	0.75	0.75	0.75
23	1.00	0.29	0.78	1.00	0.88
24	0.58	0.00	1.00	0.58	0.74
25	0.58	0.08	0.88	0.58	0.70
26	0.63	0.38	0.63	0.63	0.63
27	0.75	0.75	0.50	0.75	0.60
28	0.65	0.00	1.00	0.65	0.79
29	0.88	0.25	0.78	0.88	0.82
30	0.60	0.00	1.00	0.60	0.75
31	0.64	0.00	1.00	0.64	0.78
32	0.33	0.50	0.40	0.33	0.36
33	0.72	0.11	0.87	0.72	0.79
34	1.00	0.00	1.00	1.00	1.00
35	0.44	0.44	0.50	0.44	0.47
36	0.83	2.00	0.29	0.83	0.43
37	0.80	0.80	0.50	0.80	0.62
38	0.82	0.45	0.64	0.82	0.72
39	0.86	0.29	0.75	0.86	0.80
40	0.80	0.50	0.62	0.80	0.70
41	0.82	0.36	0.69	0.82	0.75
42	0.78	0.56	0.58	0.78	0.67
43	0.94	0.17	0.85	0.94	0.89
44	0.90	0.30	0.75	0.90	0.82
45	1.00	0.33	0.75	1.00	0.86
46	0.90	0.50	0.64	0.90	0.75
47	1.00	0.00	1.00	1.00	1.00
48	0.88	0.75	0.54	0.88	0.67
49	0.87	0.20	0.81	0.87	0.84
50	0.88	0.50	0.64	0.88	0.74
51	0.75	0.13	0.86	0.75	0.80
52	1.00	0.13	0.89	1.00	0.94
53	1.00	0.25	0.80	1.00	0.89
54	0.80	0.40	0.67	0.80	0.73
55	0.80	0.20	0.80	0.80	0.80
56	0.78	0.11	0.88	0.78	0.82
57	1.00	0.29	0.78	1.00	0.88
58	0.75	0.17	0.82	0.75	0.78
59	0.90	0.00	1.00	0.90	0.95
60	1.00	0.33	0.75	1.00	0.86
61	1.00	0.57	0.64	1.00	0.78
62	1.00	0.25	0.80	1.00	0.89
63	0.86	0.29	0.75	0.86	0.80
64	0.79	0.21	0.79	0.79	0.79
65	0.88	0.25	0.78	0.88	0.82
66	0.83	0.33	0.71	0.83	0.77
67	0.63	0.38	0.63	0.63	0.63
68	0.75	0.00	1.00	0.75	0.86
69	0.80	0.20	0.80	0.80	0.80
70	0.80	0.00	1.00	0.80	0.89
Average					0.77

**Table 3:** Average execution time of the tensor-based methods for the color test video sequences.

Method	Best rank-R [13]	HOSVD [7]	HOSVD (This Paper)
Processing time (frames/s)	3	15	160

**Table 4:** Method parameters for results presented in Table 2.

Parameter	Description	Value
*W*	Size of the tensor window used to build a model (step 1 in Algorithm 1)	47
*a*, *b*	Tensor frame fit measure (16)	*a* = 3.0
		*b* = 0.02
*G*	Number of consecutive frames to launch rebuild of the tensor model.	9
*D*	Randomization factor.	0.33
*η*	Eigenvalue fit parameter in (19).	1.1
